# The effect of choosing optimizer algorithms to improve computer vision tasks: a comparative study

**DOI:** 10.1007/s11042-022-13820-0

**Published:** 2022-09-28

**Authors:** Esraa Hassan, Mahmoud Y. Shams, Noha A. Hikal, Samir Elmougy

**Affiliations:** 1 Faculty of Artificial Intelligence, Kafrelsheikh University, Kafrelsheikh, 33516 Egypt; 2 Department of Information Technology, Faculty of Computers and Information, Mansoura University, Mansoura, 35516 Egypt; 3 Department of Computer Science, Faculty of Computers and Information, Mansoura University, Mansoura, 35516 Egypt

**Keywords:** Optimization algorithm, Stochastic gradient decent, Rung Kutta optimization, Deep ensembles, Medical images

## Abstract

Optimization algorithms are used to improve model accuracy. The optimization process undergoes multiple cycles until convergence. A variety of optimization strategies have been developed to overcome the obstacles involved in the learning process. Some of these strategies have been considered in this study to learn more about their complexities. It is crucial to analyse and summarise optimization techniques methodically from a machine learning standpoint since this can provide direction for future work in both machine learning and optimization. The approaches under consideration include the Stochastic Gradient Descent (SGD), Stochastic Optimization Descent with Momentum, Rung Kutta, Adaptive Learning Rate, Root Mean Square Propagation, Adaptive Moment Estimation, Deep Ensembles, Feedback Alignment, Direct Feedback Alignment, Adfactor, AMSGrad, and Gravity. prove the ability of each optimizer applied to machine learning models. Firstly, tests on a skin cancer using the ISIC standard dataset for skin cancer detection were applied using three common optimizers (Adaptive Moment, SGD, and Root Mean Square Propagation) to explore the effect of the algorithms on the skin images. The optimal training results from the analysis indicate that the performance values are enhanced using the Adam optimizer, which achieved 97.30% accuracy. The second dataset is COVIDx CT images, and the results achieved are 99.07% accuracy based on the Adam optimizer. The result indicated that the utilisation of optimizers such as SGD and Adam improved the accuracy in training, testing, and validation stages.

## Introduction

**Fig. 1:**
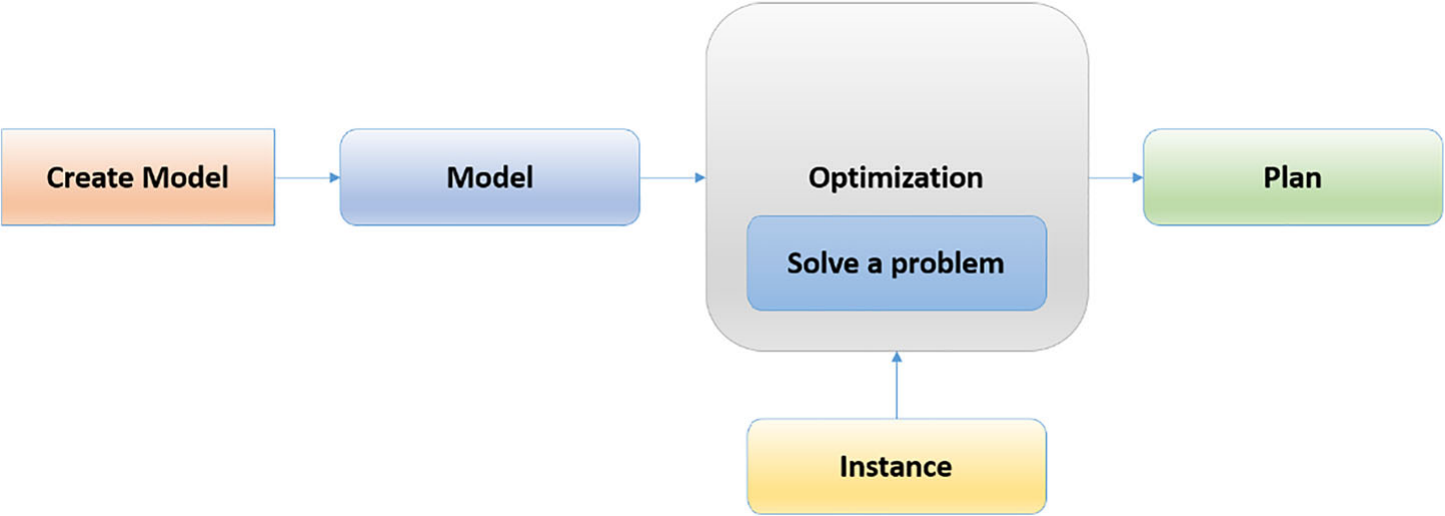
The general overview for optimization algorithm idea

Machine learning (ML) uses data and algorithms to replicate how humans learn and constantly improve its accuracy. Statistical techniques are applied to train algorithms and subsequently improve visual tasks and predict them. The data expansion task is growing, and the demand to find the most optimal solution has become widespread. Consequently, the required data have also expanded. Then, based on the input data, a data pattern is estimated using an optimization algorithm, as shown in Fig. 1 [32]. By using data, the objective function can estimate the model prediction and model accuracy. Once the model can fit the data points in the training set, weights are adjusted to reduce the distance between the known data and the model prediction [7]. Supervised learning entails the use of labeled datasets to train algorithms for predicting outcomes. As more data is introduced into the model, weights are continuously adjusted until the model is properly fitted, implying that one of the important tasks is to ensure that the model does not suffer from overfitting or underfitting [75]. Organizations use supervised learning to tackle a range of real-world problems at different scales, such as spam classification by using a distinct folder of an email account. Unsupervised learning analyzes unlabeled datasets via ML techniques. Deep learning (DL) is a popular method of addressing a variety of real-world issues. In DL, the dataset is used to train a computer, supposedly to increase its performance over time [81]. When an input value is given to the model, a function is applied to it, and it is turned into an output value through a series of layers. Thereafter, the generated output is compared with the real output, and the model calculates the difference [80]. Then, the resulting output is propagated into the model to lessen the difference. The DL architecture adjusts the weights and repeats the process until a convergence is achieved [46, 77]. An algorithm is searched to speed up the learning process while producing the best results. The main motivation behind this study is to compare with more virous optimizers to find out which one of them is best for solving medical diagnosis datasets without the need for human intervention. The algorithms can uncover hidden patterns in the data to find similarities and differences for computer vision tasks. The challenge with optimization is to identify a group of input data points for an objective function and the maximum or minimum function evaluation points. Several optimization techniques have been created and tested in this direction of solving a variety of problems. The impact of the most extensively used optimization algorithms on the learning process is investigated in this survey [89]. ML and DL are used as optimization methods to learn the parameters of the input data [82]. In particular, the parameters of the input data are learned via ML and DL as the optimization methods. The researchers of this study view optimization techniques as critical in successfully implementing real-world solutions [59].

ML optimization is the process of altering the hyperparameters to minimize the cost function using a certain optimization approach. The cost function must be minimized because of its specific task of determining the difference between the true value of an estimated parameter and the value predicted by the model [13]. However, prior to this task, the model parameters must be distinguished from the hyperparameters. In addition, prior to the training of the model, the hyperparameters must be specified. The number of clusters, learning rate (LR), among others, should be considered. A model’s structure is described by its hyperparameters. However, the model’s parameters can only be obtained during the training. At present, no existing method can calculate the parameters ahead of time.

Similarly, the model’s weights should be known in advance, but this task continues to be a challenge. Currently, trial and error are adopted with the loss function, and optimizers use the result to determine the ways of altering a neural network’s weights or LRs to reduce the loss [90]. Optimization algorithms are used to minimize the losses and ultimately deliver the most precise outcomes to the best extent possible. The process normally starts by defining a loss function for a DL problem. An optimization procedure is applied to minimize the loss after the loss function is obtained [60]. A loss function is frequently referred to as the optimization problem’s objective function during optimization. In history and practice, the majority of the optimization algorithms have focused on minimization. Meanwhile, a straightforward method of maximization is to simply reverse the sign on the objective. Although optimization contributes to DL by lowering the loss function, the goals of optimization and DL are fundamentally different [64]. Optimization is focused on minimizing an objective, whereas DL is oriented towards finding a good model given a finite amount of data. Moreover, the training error and the generalization error differ from each other, as the objective function of an optimization algorithm usually depends on a loss function based on the training dataset, in which the goal of the optimization is to reduce the training error [87]. Location problem, in example, may take into account a number of distinct (and potentially competing) objectives, such as obtaining a level of service commensurate to the location’s importance, lowering the worst-case service level, and raising the average service level. Taking into account all those goals in a single mathematical problem could result in a great number of answers that confound the decision-maker rather than aid them. Due to this, our study offers a novel analysis based on the comparison of various location solution characteristics using a battery of key performance indicators (KPIs). We also examine the trend of the given KPIs over the interventions to produce long-term managerial insights, since charging infrastructures are often expected to be located through a series of progressive interventions over a predetermined time [29, 30]. By contrast, DL aims to reduce the generalization errors. To achieve the latter, the overfitting and the optimization procedure must be both considered when lowering the training error. Rather than focusing on the generalization error of the model, the emphasis is on the performance of the optimization techniques for minimizing the objective function. The majority of the objective functions in DL are complex and devoid of analytical solutions. Thus, numerical optimization algorithms must be used instead. All of the optimization algorithms discussed in this paper fall into the DL category. Nonetheless, DL optimization is fraught with difficulties. Local minima, saddle points, and disappearing gradients are among the most perplexing issues. For example, the DL models’ objective functions frequently have plenty of local optima. As the gradient of the objective function’s solutions approaches or becomes zero, the numerical solution found by the final iteration can only minimize the objective function locally rather than globally. This issue is apparent when the numerical solution of an optimization problem approaches the local optimum. Only a small amount of noise allows for the parameter to leave the local minimum [53]. In reality, the natural change of the gradients in mini-batches can dislodge the parameters from the local minima. This practical concern is one of the advantages of the mini-batch stochastic gradient descent (SGD) [32]. This study offers the following contributions:

The methods of selecting optimization algorithms in computer vision tasks are comprehensively surveyed.

- The motivations for using optimization algorithms to improve computer vision tasks are summarized.
- The open challenges pertaining to the effects of optimization algorithms in computer vision tasks are investigated.
- The effects of the selected algorithms on the final result are compared on the basis of measure metrics.

The rest of the comparative study is organized as follows. Section 2 describes the optimization algorithms. Section 3 presents a case study for skin cancer diagnosis. Section 4 concludes the survey.

## Optimization algorithms

**Fig. 2:**
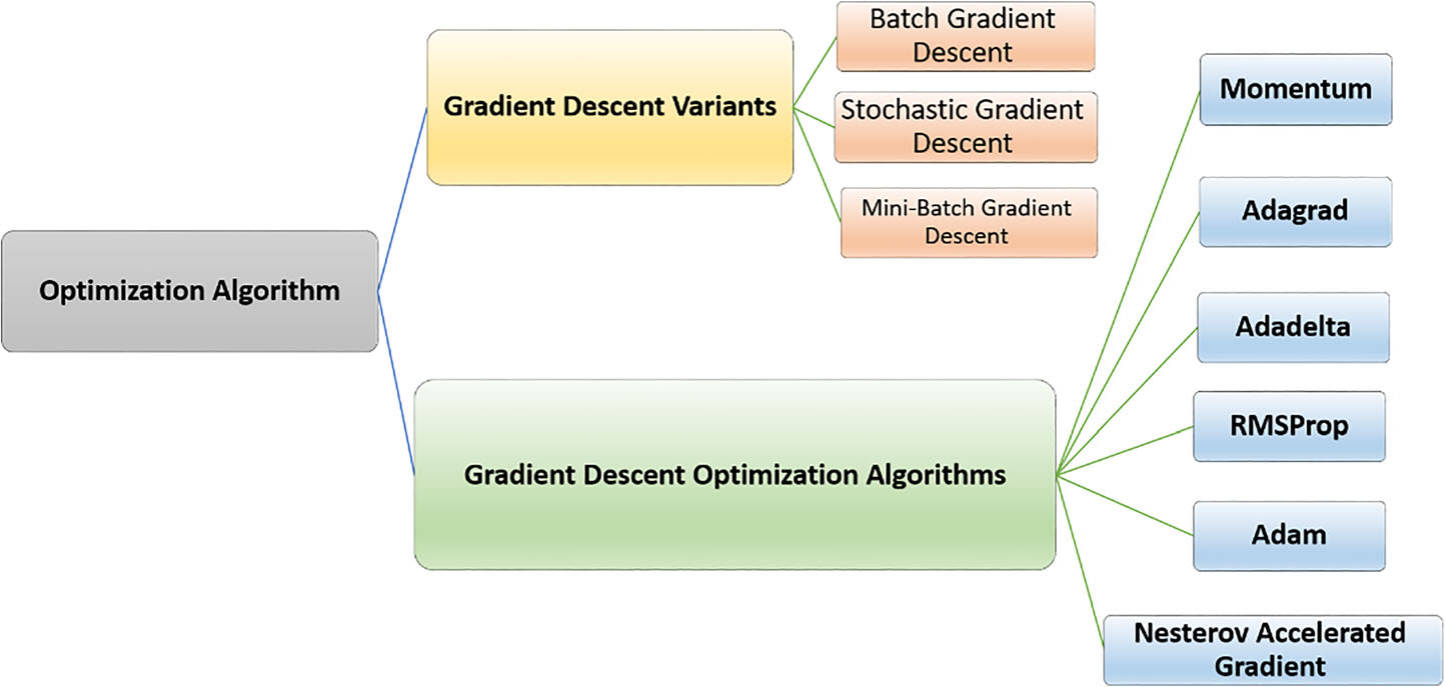
The general structure for optimization algorithms

Optimization algorithms are the foundation on which a machine learns from its mistakes. Gradients are calculated, and the loss function is reduced to the smallest possible value. Learning can be implemented in many ways using optimization techniques, as shown in Fig. 2 [7, 68, 75]. The algorithms selected in this study are presented in the next sections. In this study, we highlighted the most common optimization algorithms such as gradient decent variants and gradient decent optimization. The gradient decent variant is generally categorized to batch gradient decent, stochastic gradient decent and mini-batch gradient decent. While the gradient decent optimization algorithms can be classified to momentum, Adagrad, Adadelta, RMSProp, Adam and Nestrov accelerated Gradient. The utilization of SGD, minibatch gradiend decent are more helpful to handle the over-fitting problem as well as optimization problem to boost the evaluation accuracy [26]. Moreover Adam optimizer are most commonly used to handle the medical images [70].

### Gradient descent algorithm

Neural network algorithms are improved by taking a small batch of data and performing a type of gradient descent on them. The gradient descent calculates the slope of the landscape, which is the derivative of the function at this point with respect to the weights, as shown in Eq. (1) [75].

1 $$w=w- lr.{\nabla}_wL(w)$$

A constant value is adopted for the LR to determine the step size at each iteration as the calculation moves towards a minimum loss function [7, 81]. SGD is a fast and computationally efficient approach, but it adds noise in the estimation of the gradient. The frequent updating of the weight can lead to large oscillations, causing the training process to be extremely unstable. A list of stochastic optimization techniques is shown in the succeeding subsections, and each technique can be updated on a regular basis. The Gradient Descent has many advantages, such as easy computation, implementing, and understanding. It has some defects, such as weights being changed after calculating the gradient on the whole dataset. So, if the dataset is too large, it may take years to converge to the minima. It requires large memory to calculate the gradient on the whole dataset.

#### Stochastic gradient descent (SGD)

SGD is a basic algorithm and widely used in ML algorithms. Instead of calculating the gradient over all training examples and updating the weights, the SGD updates the weights of each training example *x*_*i*_, *y*_*i*_, as shown in Eq. (2) [32].

2 $$w=w- lr.{\nabla}_wL\left({x}_i,{y}_i,W\right)$$

**Fig. 3:**
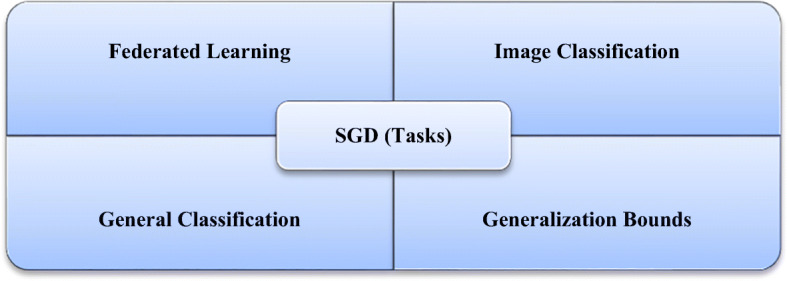
SGD optimizer algorithm tasks in several computer vision problems

**Fig. 4:**
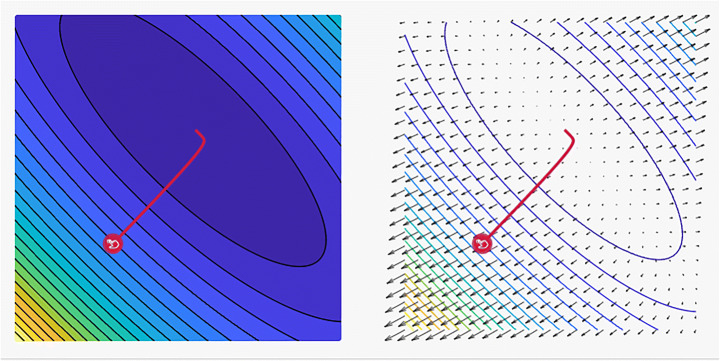
A plot of the loss reveals distinct properties for SGD optimizer with its style of convergence by ensample visualization tool, where the steps that the optimizer takes plotted in red, Coordinates: (6.00, 14.00), Global Minimum: (1, 3), Optimizer Minimum: (1.034, 2.966) [18, 85]

The central idea is to start with a random point, and then a technique for updating is selected for each iteration as they descend the slope. The SGD method randomly selects a single data point from the entire dataset at each iteration to ease the computation. In “mini-batch” gradient descent, which is considered a common technique, a small number of data points instead of only one data point is sampled at each step [7]. However, this basic version of the SGD has certain limitations that can negatively affect the training. If the change in the loss function is fast in one direction and slow in another, then the oscillation of the gradients will be high, rendering the training progress to be extremely slow [32]. Furthermore, if the loss function has a local minimum, then the SGD will likely be stuck, and a good minimum cannot be determined. These problems occur when the gradient reaches zero and the weight or other relevant parameters are not updated. The gradients are noisy because they are estimated on the basis of only a small sample of the dataset. Subsequently, the noisy updates may not correlate well with the true direction of the loss function [75]. Selecting a good loss function is challenging and requires time-consuming experimentation with different hyperparameters. The same LR is applied to all parameters, which is problematic for features with different frequencies or significant attributes. Many improvements have been proposed over the years to overcome some of the aforementioned issues. Figure 3 shows the main and common tasks of the SGD optimizer in which federated learning and image classification have the highest precision among all of its performed tasks [81]. Figure 4 shows a plot of the loss, revealing the distinct properties of the SGD optimizer and its style of convergence in a specific coordinate.

Wenzel et al. [80] demonstrated that the posterior predictive created by the Bayes posterior produces systematically inferior predictions compared with the simpler approaches, such as the point estimates provided by SGD via the Markov chain Monte Carlo sampling method. Numerous theories have been proposed to explain the cold posterior effect, and predictions have been tested by experiments. Their research has casted doubt on the goal of correct posterior approximations of the Bayesian DL. Noroozi et al. [57] suggested a model for the Schema-Guided Dialogue dataset, which includes natural language descriptions for all elements. Table 1 presents some common tasks that use the SGD optimizer algorithm. According to a previous study, increasing the batch size of the SGD does not change the expectation of the stochastic gradient, but the variance is reduced. When the batch size is large, LR can be increased to achieve the opposite direction of the gradient. In general, SGD plays an important role in computer vision tasks, but it has not yet solved the two major problems associated with gradient descent. Thus, SGD is often combined with other algorithms, such as Momentum and AdaGrad; these algorithms will be presented in the following sections. Using the SGD has a number of advantages, including frequent changes in the model parameters, indicating a much more rapid convergence. The values of the loss functions can also be ignored, suggesting less memory usage, and a new minimum may also be derived. Nonetheless, SGD entails certain limitations, such as excessive variance in the model parameters. Even after attaining the global minima, the algorithm may continue to burn. For the SGD to achieve the same convergence as that in gradient descent, the LR must be gradually reduced.

**Table 1:** Some Related works for using SGD optimizer with vision datasets

Year	Author	Tasks	Dataset	Metrics
2021	Wenzel et al. [80]	Image Classification	CIFAR10 SVHN	ACC = 88.2%
2021	Li et al. [46]	Limited bandwidth TCP interconnects network	GLUE	ACC = 83.9%
2021	Tang et al. [77]	TCP interconnects	Celeb A GLUE	ACC = 83.9%
2020	Noroozi et al. [57]	Dialogue state tracking	Data Augmentation Goal-Oriented Dialogue	ACC = 95.70%

SGD has not yet solved two major disadvantages of gradient descent. As a result, SGD is combined with other algorithms such as Momentum and Ada Grad. These algorithms will be presented in the following sections.

It has several advantages, including frequent changes of model parameters, which means it converges faster. Hence, there is no need to keep the values of loss functions; hence, it uses less memory. It’s possible that it’ll acquire new minima as well. SGD has some flaws, such as excessive model parameter variance. Even after attaining global minima, it may continue to burn. To achieve the same convergence as gradient descent, the learning rate must be gradually reduced.

#### SGD with momentum

In this approach, a momentum term is added to the regular SGD to overcome the limitations of the gradient descent algorithm, i.e., a gradient descent with a momentum. By using the principle of momentum from physics, the SGD is forced to continue moving in the same direction as those in the previous time steps. This momentum is accomplished by introducing two new variables, namely, velocity and friction, as given by Eqs. (3) and (4), respectively [46].

3 $$vt+1= pvt+\nabla wL\left(x,w\right)$$

4 $$w=w- lr. vt+1$$

Velocity is computed as the running mean of the gradients up to a certain point in time, indicating the direction to which the gradient should keep moving. Friction is a constant number for achieving decay. At each time step, velocity is updated by decaying the previous velocity by a factor and adding the gradient of the weights at the current time. Then, weights are updated in the direction of the velocity vector.

**Fig. 5:**
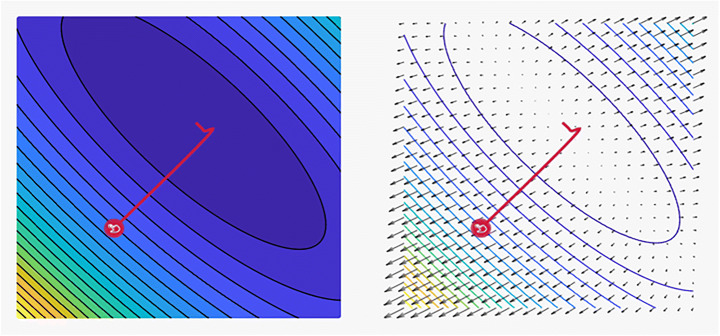
A plot of the loss reveals distinct properties for SGD with Momentum optimizer with its style of convergence by ensample visualization tool, where the steps that the optimizer takes plotted in red, Coordinates: (6.00, 14.00) Global Minimum: (1, 3)and, Optimizer Minimum: (1.023, 2.977) [18, 85]

Radosavovic et al. [65] investigated the ResNet design space and found the network design to contradict practice. The ResNet design area offers simple and fast networks that perform well in a variety of failure regimes. ResNet models outperform the popular EfficientNet models in similar training settings and are five times quicker when solved on GPUs. Author in [93] proposed a modularized architecture that uses channel-wise attention on multiple network branches to improve the ability to capture cross-feature interactions and learn diverse representations. In their work, the unified and simple calculation block can be specified using only a few variables. Furthermore, in [52] adopted an architecture with a simple and unified computing block that may be parameterized with only a few variables. The pre-trained model can outperform EfficientNet in terms of accuracy and latency tradeoff during image classification. Resent has also been adopted in the winning submissions of the COCO-LVIS challenge, and superior transfer learning outcomes on multiple public benchmarks acting as the backbone are achieved. Table 2 presents some of the tasks that commonly use SGD with the Momentum optimizer algorithm. Figure 5 shows a plot of the loss, revealing the distinct properties of the SGD with Momentum optimizer and its style of convergence in a specific coordinate. As for the scale-decreased backbone, Du et al. [24] proposed that the encoder–decoder architecture can be ignored when creating strong multi-scale features. SpineNet is a backbone comprising scale-permuted intermediate characteristics and cross-scale connections, which are learned by applying the neural architecture search (NAS) method on an object detection problem. Khosla et al. [42] investigated two different variants of the supervised contrastive loss to determine which one is the most effective. The top-1 accuracy is 81.4% on the ImageNet dataset with ResNet-200, a value that is 0.8% higher than the best value recorded for this architecture. On other datasets and two ResNet variations, the cross-entropy is consistently surpassed. The loss presents advantages in terms of natural corruption resistance, and it is relatively stable in terms of handling the hyperparameter settings, such as optimizers and data augmentations [27, 71, 73]. Moreover in [66] SGD with momentum optimizer of the applied ImageNet dataset were presented in classification stage achieved loss rate 37.1%. In [23] MNIST dataset with CIFAR-10 are further applied based on SGD to boost the classification proce.

**Table 2:** Some Related works for using SGD with Momentum optimizer with vision datasets

Year	Authors	Tasks	Datasets	Metrics
2020	Zhang et al. [93]	• Image Classification • Semantic Segmentation • Transfer Learning	• ImageNet • COCO Cityscapes • ADE20K PASCAL	Accuracy = 81.13%
2020	Du et al. [24]	• Image Instance Segmentation • Real-Time Object Detection	• ImageNet • COCO • I Naturalist	AP = 52.5%
2020	Khosla et al. [42]	• Image Classification • Data Augmentation	• ImageNet	Accuracy = 78.7%
2020	Radosavovic, et al. [66]	• Image Classification	• ImageNet	Loss = 37.1%
2021	Ding et al. [23]	• Image Classification	• CIFAR-10 • ImageNet • MNIST	Accuracy = 80%

Gradient descent does not reach the global minimum point; instead, it merely reaches the local minimum point. By contrast, SGD with Momentum assists the ball in crossing the inclined space prior to reaching its destination. However, as the ball moves nearer to the target, the calculation takes a long time to handle the slope variations before completely halting. This phenomenon can be explained by the marble’s momentum. This algorithm has several advantages, including the ability to reduce oscillations and its high variance in handling the parameters, and it can converge faster than gradient descent. Its disadvantages include the addition of an extra hyperparameter that must be specified manually and precisely.

### Rung Kutta optimizer

**Fig. 6:**
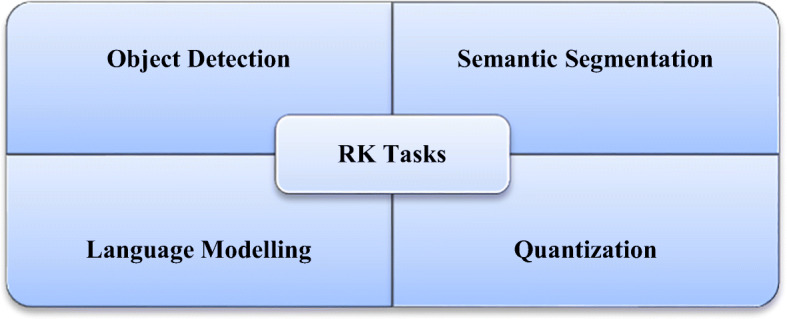
RK optimizer algorithm tasks in several computer vision problems

**Fig. 7:**
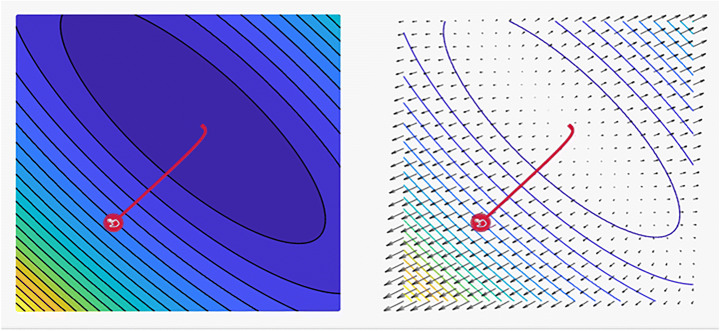
A plot of the loss reveals distinct properties for Rung Kutta optimizer optimizer with its style of convergence by ensmallen visualization tool, where the steps that the optimizer takes plotted in red, Coordinates: (6.00, 14.00)Global Minimum: (1, 3)and, Optimizer Minimum: (1.426, 2.568) [18, 85]

The Rung Kutta (RK) optimizer can address a wide range of future optimization challenges. As a promising and logical global optimization search process, the RK optimizer employs the logic of slope variations. The RK optimization process is shown in Fig. 6 and the common tasks that use this technique. When examining the prospective regions of a feature space, with the aim of reaching the global optimum, this search strategy benefits from two active stages, namely, exploration and exploitation. The efficiency of the RK algorithm was compared with the efficiency of other metaheuristic algorithms by considering 50 mathematical test functions and four real-world engineering situations [1]. RK optimization can provide promising and competitive outcomes given its superior exploration and exploitation stages, fast convergence rate, and avoidance of the local optima. Nonetheless, the suitability of this deep-rooted optimizer as a tool for real-world optimization should be evaluated [1, 9]. Figure 7 shows a loss plot revealing the distinct properties of the Rung Kutta optimizer and its style of convergence in a specific coordinate.

The Big DL framework, which has been utilized by a range of users in the industry for building DL models, was studied by Jason Dai et al. [19]. DL applications can run on an Apache Hadoop cluster to directly process production data and partake in the deployment and management pipeline for end-to-end data analysis. Real-world trends from using Big DL have been published in the past. Xu et al. [83] predicted facial box and landmark positions in real time with high accuracy. Their proposed method can be classified as an anchor-free approach. Their work was accomplished by learning the bounding box of each position potentially containing a face, from which semantic maps were adopted for each position. Ding et al. [23] described a convolutional neural network (CNN) architecture with a VGG-like inference-time body composed of a stack of 3 × 3 convolution and ReLU and a multi-branch training-time model. A structural re-parameterization technique was applied to decouple the training and inference time of the architecture; the model is appropriately called the Rep VGG. The accuracy of this approach is over 80% on ImageNet, which is the first time for a straightforward model to obtain this rate. Hoffman et al. [36] suggested a reinforcement learning algorithm created in academic and corporate labs. Baseline implementations composed of several algorithms were built with the available framework. The primary design considerations were ignored; instead, the focus was on Acme and how it could be leveraged to create the baselines. The agents at various levels were tested in terms of complexity and computation ability, including the related distributed versions. Table 3 lists some of the tasks that commonly use the RK optimizer algorithm.

**Table 3:** Some common related works for using RK optimizer algorithm

Year	Author	Tasks	Dataset	Metrics values
2021	Ding et al. [23]	• Image Classification • Semantic Segmentation	• CIFAR-10 • ImageNet • MNIST • Cityscapes	ACC = 78.5%
2018	Jason Dai et al. [19]	• Fraud Detection • Object Detection	• ImageNet	N/A
2019	Xu et al. [83]	• Face Detection	• Wider face dataset	ACC = 0.935%
2020	Matt Hoffman et al. [36]	• DQN Replay Dataset • Offline RL	• DQN Replay Dataset	N/A

### Adaptive learning rate (AdaGrad)

**Fig. 8:**
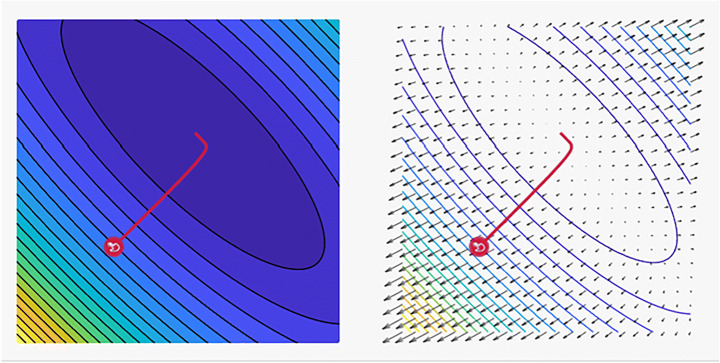
A plot of the loss reveals distinct properties for Adagrad optimizer with its style of convergence by ensmallen visualization tool, where the steps that the optimizer takes plotted in red, Coordinates: (6.00, 14.00)Global Minimum: (1, 3)and, Optimizer Minimum: (1.018, 2.982) [28]

AdaGrad performs small updates on frequently used features and large updates on infrequently used features. This algorithm can overcome some of the issues encountered by SGD. AdaGrad is a technique of adjusting the LR according to the parameters as shown in Fig. 8. The parameters linked to the frequently occurring features are slightly adjusted, whereas the parameters linked to the infrequently occurring features are updated, indicating a variation in the LRs. The root of the squared gradients and the magnitude of the gradients are both considered. AdaGrad, an optimization approach of the AdaGrad family, was introduced by Defazio et al. [22] as in Eq. 5.

5 $$w=w-\frac{1}{\sqrt{G}+e}\kern0.5em \odot {\left(\nabla w\kern0em L\left(x,y,W\right)\right)}^2$$

where $G={\sum}_{t=1}^T\nabla w\kern0em L\left(x,y, Wt\right)$ such that the AdaGrad outperforms the other DL optimization algorithms in a variety of disciplines, including vision classification and image-to-image tasks. Even on issues in which adaptive methods typically perform poorly, AdaGrad can match SGD and Adaptive Moment (Adam) on the test sets of certain tasks. According to [31] recall and precision enhancements are the two effective options in AdaGrad, and they can be integrated into the end-to-end network. The method called the Corner Proposal Network (CPN) can detect objects of varied sizes while also avoiding being misled by many false-positive suggestions. CPN has an AP of 49.20% on the MS-COCO dataset and is competitive with state-of-the-art object detection algorithms. Different from the first-order methods (e.g., SGD and Adam), the second-order algorithms are among the most powerful optimization algorithms entailing superior convergence features. In an unsupervised domain adaptation (UDA) setting, Yao et al. [86] described a strategy for encoding visual task correlations to boost model performance. Semantic segmentation and monocular depth estimation were proven to be complementary tasks, and the appropriate encoding of their links increased the performance on both tasks in a multitask learning scenario. According to Chen et al. [17], CADA is a collection of new rules optimized for adaptive stochastic gradients, and it can be implemented to save on communication upload. The new methods adaptively reuse stale Adam gradients, conserving communication while maintaining similar convergence rates to the Adam optimizer. Table 4 shows some of the tasks that commonly use the AdaGrad optimizer algorithm. A notable drawback of AdaGrad is the decreasing LR over time because of the monotonic increment of the running squared sum. Nonetheless, one of the most obvious advantages of AdaGrad is that it eliminates the need to manually modify the LR. By simply setting the default learning speed to 0.01, the algorithm can then adjust itself. AdaGrad’s disadvantage is that the variable sum of squares increases over time, causing the learning pace to become extremely slow and the training to freeze.

**Table 4:** Some common related works for using AdaGrad optimizer algorithm

Year	Author	Tasks	Dataset	Metrics Values
2020	Yao et al. [86]	• Stochastic Optimization	WikiText-103	ACC = 93.08%
2020	Chen et al. [17]	• logistic regression task	CIFAR10	ACC = 95%
2021	Defazio et al. [21]	• Stochastic Optimization	CIFAR-10	ACC = 94.15%
20,220	Duan et al. [63]	• Object Detection	COCO	N/A
2020	Saha et al. [69]	• Estimation Semantic Segmentation • Estimation Multi-Task Learning • Unsupervised Domain Adaptation	Cityscapes SYNTHIA Virtual KITTI	N/A

### RMSProp optimizer

The sizes of gradients vary by weight and change over time, hence the difficulty in selecting a single global LR. This aspect is addressed by RMSProp by retaining a moving average of the squared gradient and altering the weight updates by this magnitude. The gradient updates are elaborated in [43] as shown in Eqs. 5 and 6.

6 $$vt={\delta}_{vt-1}+\left(1-\delta \right){\left({\nabla}_wL\left(x,y,{w}_t\right)\right)}^2$$

7 $$w=w-\frac{1}{\sqrt{v_t}+e}\odot {\left({\nabla}_wL\left(x,y,w\right)\right)}^2$$

Table 5 presents some of the related works, including that of Khosla et al. [42] who compared two different supervised contrastive loss models. The top-1 accuracy of 81.4% is achieved on the ImageNet dataset with ResNet-200, a value that is 0.8% higher than the best value recorded for this architecture. On the other datasets and two random variations, the cross-entropy can be consistently surpassed. A semi-supervised learning algorithm presented by Pham et al. [62] can achieve an accuracy of 90.29% on ImageNet. Meta Pseudo-Labels (MPLs) use a teacher network to instruct a student network by generating pseudo-labels on unlabeled input. Different from Pseudo-Labels in which the teacher is fixed, the teacher is continually adapted in MPL via the feedback of the student’s performance on the labeled dataset. Various measurements of efficiency on different hardware platforms and a wide range of application scenarios were considered by Graham et al. [34]. Tests were performed to experimentally support the technical choices in their study, eventually determining their approach to be applicable on a majority of systems. The accuracy is 80% on ImageNet. The EvoNorms, a series of innovative normalization activation layers with architectures surpassing the established design patterns, were discovered by Liu et al. [48]. The feature maps were centered on the activation functions. Their tests showed that EvoNorms could outperform different image classification models, such as ResNet and Mask R-CNN with SpineNet, for the image synthesis of segmentation-based layers. The denominator is the root mean square (RMS) error of the gradients, hence the name of the algorithm. In most adaptive rate algorithms, a very small value denoted by e is added to prevent the nullification of the denominator. Usually, e is equal to 1e-7. The most obvious benefit of using RMSprop is that it solves AdaGrad’s problem of progressive learning pace (i.e., decreasing learning speed over time, thus slowing down the training, possibly leading to freezing). As for the drawback, the RMSprop algorithm can only calculate the local minimum rather than the global minimum (i.e., Momentum) [12]. The two momentum algorithms can be integrated with RMSprop to create an optimal Adam algorithm, as to be discussed in the next section.

**Table 5:** Some common related works for using RMSProp optimizer algorithm

Year	Authors	Tasks	Datasets
2020	Khosla et al. [42]	Contrastive Learning Representation Learning Data Augmentation Image Classification Self-Supervised Learning	ImageNet ImageNet-C
2021	Pham et al. [62]	Image Classification Meta-Learning Semi-Supervised	CIFAR-10 ImageNet SVHN
2021	Graham et al. [34]	Image Classification	CIFAR-10 ImageNet
	Liu et al. [48]	Image Classification Image Generation Instance Segmentation Semantic Segmentation	CIFAR-10 COCO

On the other hand, the denominator is the root mean squared error of the gradients (RMS), hence the name of the algorithm. In most adaptive rate algorithms, a very small value e is added to prevent nullification of the denominator; usually, it is equal to 1e-7.

### Adaptive moment estimation (Adam)

**Fig. 9:**
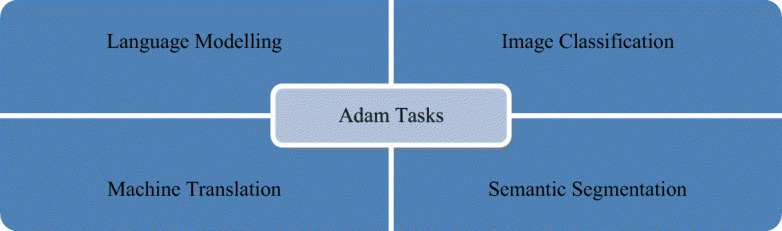
Adam optimizer algorithm tasks in several computer vision problems

Adam is a first-order gradient-based optimization technique for stochastic objective functions based on adaptive lower-order moment estimates. Instead of using the usual SGD approach, Adam is used to iteratively update the network weights depending on the training data as shown in Fig. 9. Adam stems from evolutionary moment calculation and algorithm features as shown in Eqs. (7), (8), and (9).

8 $${m}_t={\delta}_{mt}+\left(1-{\delta}_1\right)\left({\nabla}_wL\left(x,y,{w}_t\right)\right)$$

9 $${v}_t={\delta}_{2 vt-1}+\left(1-{\delta}_2\right){\left({\nabla}_wL\left(x,y,{w}_t\right)\right)}^2$$

10 $$w=w- lr.\frac{m_t}{\sqrt{v_t+e}}$$

**Fig. 10:**
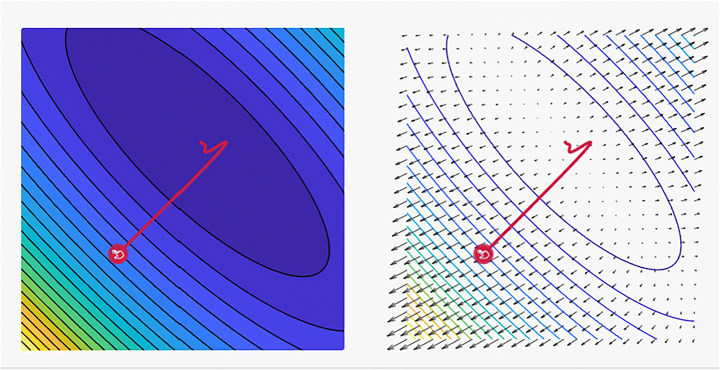
A plot of the loss reveals distinct properties for Adam optimizer with its style of convergence by ensmallen visualization tool, where the steps that the optimizer takes plotted in red, Coordinates: (6.00, 14.00)Global Minimum: (1, 3)and, Optimizer Minimum: (1.022, 2.976) [28]

It has many features and is the most common and fastest optimizer for ML techniques. The advantages of using Adam include its (i) easy implementation and (ii) efficient computing, and (iii) memory requirements are not needed. Adam can be viewed as a combination of RMSprop and momentum. The Adam algorithm has grown in popularity over the years, and efforts have been pursued to further optimize this technique. The two most promising variations of Adam are the AdaMax and Nadam, which are supported by deepest-learning frameworks. Xin et al. [82] found this scheme to have a sluggish inference speed, hence the difficulty of using it in real-time applications. Aiming to speed up the BERT inference, DeeBERT, which is a simple but effective approach, was subsequently considered. This method allows for the samples to quit the model at a much earlier time without having to undergo a complete process. Experimental results suggest that DeeBERT can reduce inference time by up to 40% without compromising model quality. Furthermore, the examinations were able to demonstrate the various behaviors in the BERT transformer layers and their redundancy. Consequently, new ways of using deep transformer-based models were recommended to solve downstream problems. Mobile BERT is a method proposed by Sun et al. [76] to compress and speed up the popular BERT model. Mobile BERT, like the original BERT, is a task-agnostic technique and thus may be applied generically to various downstream NLP jobs with a slight fine-tuning. A specifically developed instructor model, namely, the inverted-bottleneck that includes the BERT LARGE model, is initially trained prior to using the Mobile BERT. Mobile BERT is 4.3 times smaller and 5.5 times faster than the BERT BASE according to empirical investigations, and it can attain competitive results on well-known benchmarks with an F1 score of 90%. Akbari et al. [2] showed that the convolution-free VATT outperforms the state-of-the-art convent-based designs in downstream tasks. VATT’s vision transformer has achieved new performance marks (82.1% accuracy on Kinetics-400, 83.6% accuracy on Kinetics-600, and 41.1% accuracy on Moments in Time) while avoiding supervised pre-training. On ImageNet, the transfer-to-image classification result is 78.7% (top-1 accuracy), superseding the transfer-from-scratch result of 64.7% when training on the same Transformer, hence demonstrating the generalizability of their model despite the domain mismatch between videos and images.In the fields of cross-lingual classification and unsupervised and supervised machine translation, Lample et al. [44] achieved state-of-the-art results. On XNLI, the technique can improve state-of-the-art configurations by 4.94% in terms of absolute accuracy. On the WMT16 German–English, 34.3 BLEU was attained via unsupervised machine translation, outperforming the prior state-of-the-art methods by more than 9 BLEU. On the WMT16 Romanian–English, a new state-of-the-art 38.5 BLEU for supervised machine translation was achieved. This scheme can outperform the previous best approach by more than 4 BLEU. Table 6 lists some of the most frequently encountered Adam optimizer algorithm-related tasks. As previously stated, Adam is a mix of Momentum and RMSprop. Thus, if Adam is assumed to be an extremely heavy ball with friction, then momentum is the ball that plunges downhill, quickly moving from the local minimum to the global minimum; however, the global minimum cannot be reached. Furthermore, as oscillation around the target takes a long time to complete due to friction, the algorithm may also easily stop as shown in Fig. 10.

**Table 6:** Some common related works for using Adam optimizer algorithm

Year	Author	Tasks	Datasets
2020	Sun et al. [76]	Natural Language Inference Question Answering Transfer Learning	• SQuAD • SST • MRPC • MobileBERT
2021	Akbari et al. [2]	Action Classification Action Recognition Action Recognition in Videos Self-Supervised Learning	• ImageNet • UCF101 • Kinetics
2018	Lample et al. [44]	Language Modelling Unsupervised Machine Translation	• GLUE • MUSE

### Deep ensembles (DE)

**Fig. 11:**
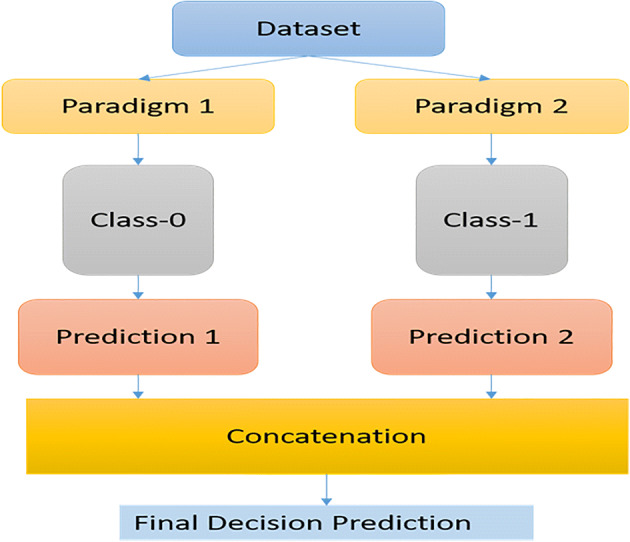
General Deep Ensembles Idea

Ensemble learning combines several individual models to improve generalization performance. Although the method requires hyperparameter tuning, it is well-suited for large-scale distributed data and can be readily implemented in a wide variety of architectures, such as CNN and those models that do not use dropouts [25] as investigated Fig. 11. Non-Bayesian approaches and other less popular evaluation metrics were recommended for the predictive uncertainty of deep ensembles. Izmailov et al. [38] suggested using a posterior representation comparable to multiple short chains. The performance of Bayesian neural networks was unaffected by the prior scale, and the results were similar for diagonal Gaussian and mixtures of Gaussian. Nonetheless, less costly alternatives, such as deep ensembles (DEs), can enhance the generalization much further when a weight normalization step is added during training, followed by a substitution of the output layer with a Gaussian process. Ahmadianfar et al. [1] recommended a model to improve the distance-awareness abilities of modern deep neural networks (DNNs). With the use of a set of vision tasks, the scheme is competitive with the DE in terms of making predictions. Basak et al. [9] generated and assembled simplicial complexes that outperformed the separately trained DEs in terms of accuracy and robustness to changes in datasets. A pre-trained model was utilized, and the method only required a few training epochs to determine the low-loss simplex. Ritter et al. [67] expanded Matheron’s conditional Gaussian sampling rule to achieve a fast weight sampling. This scheme allowed the inference technique to run faster than ensembles. More importantly, by using fully connected neural networks and ResNets, competitive performance was achieved with respect to the state-of-the-art models in terms of prediction and uncertainty estimation tasks, and the parameter size was decreased to 24.3% of that of the single neural network. Siems et al. [74] used multiple regression models on a dataset and built surrogates via DE to model the uncertainties. The merits of using a surrogate benchmark over a tabular one was also determined. The NAS-Bench-301 dataset can be used to acquire results equivalent to that of the true benchmark for a fraction of the cost. Furthermore, as the training is easily parallelized, separate networks can be considered. Explicitly decorrelating network predictions similar to the approach in Ref. [61] may enhance ensemble diversity and performance. An adaptive mixture of experts can increase the performance even further by optimizing the ensemble weights. Implicit ensembles may also be considered when members of the ensemble share the same parameters. Ensembles generally have a relatively high prediction accuracy, and the size of the ensemble affects the test results. Furthermore, ensembles can overcome the common challenges of other techniques. Nonetheless, each approach has its own unique features. For instance, during data wrangling and tweaking, various models can be tweaked to improve the fitting. As for the disadvantage, ensembles are difficult to interpret. Even the best ideas attained via the ensembles are not always able to persuade decision-makers, and they are not always adopted by the end-consumers. Finally, creating, training, and deploying ensembles is more expensive than the other methods. The return on investment of the ensemble technique should be carefully studied, as increasing the complexity is not always a good approach [11, 49] as shown in Table 7. Furthermore, method based on data aggregation to predict citywide population movements using dynamic spatiotemporal correlations [4]. Hence, utilizing Spatio-Temporal Patterns and Deep Hybrid Neural Networks to Predict Citywide Traffic Crowd Flows is presented by Ali et al. [3]. However, using attention-based neural networks to predict citywide traffic flow by using dynamic spatiotemporal correlations and convolutional neural networks with dynamic spatiotemporal graphs to forecast citywide traffic patterns [5, 6].

**Table 7:** Some common related works for using Deep Ensembles optimizer algorithm

Year	Authors	Tasks	Datasets
2021	Izmailov et al. [38]	Data Augmentation Variational Inference	IMDb Movie Reviews
2021	Ritter et al. [67]	Deep Neural Network	CIFAR-100
2020	Siems et al. [74]	Neural Architecture Search	CIFAR-10 NAS-Bench-101
2020	Liu et al. [49]	language understanding	SVHN
2021	Benton et al. [11]	Connecting Volumes	CIFAR-10

### Feedback alignment

By comparing the simple domain and demanding robot simulation tasks, Zhang et al. [94] empirically demonstrated the benefit of the suggested algorithms and their nonlinear variations via the competing density-ratio-based approach. Feedback Alignment (FA) assumes the presence of a global feedback path, which may be biologically implausible because the feedback layer needs to travel a long physical distance. The principle of FA is centered on driving the error signal. In the alignment stage, a layer cannot learn unless its upper layers are roughly aligned. Bass et al. [10], who developed the human connectome project, used UK Biobank datasets. Their method was validated via Mini-Mental State Examination cognitive test score prediction for Alzheimer’s disease The neuroimaging initiative cohort and brain age prediction for both neurodevelopment and neurodegeneration were considered. The generated FA maps could help to explain the outlier predictions, enabling the regression module to enhance the latent space disentanglement. Batch normalization, which adds implicit contrastive terms, was leveraged by Kefato et al. [41]. Then, four feature augmentation (FA) strategies for the graphs were implemented, as data augmentation is critical in contrastive learning. Even if the graph’s topological augmentation (TA) was widely employed, their empirical data showed that FA is as competitive as the TA. The model proposed by Najafi et al. [55] suggested a parallelizable model can simultaneously handle several data points. The semantic similarities of two tweets were compared. As opposed to the existing approaches, the suggested strategy was found to be effective. Kamran et al. [40] trained a generative adversarial network utilizing multiple weighted losses on separate data modalities via a semi-supervised technique. According to the tests, the proposed design can outperform the previously reported generative networks in terms of fundus-to-angiography synthesis. Furthermore, their vision transformer-based discriminators for retinal illness prediction can be generalized well on out-of-distribution datasets. Table 8 presents some of the tasks that commonly use the FA optimizer algorithm. FA requires a thorough investigation of its asymmetric outcomes under certain network assumptions.

**Table 8:** Some common related works for using Feedback Alignment optimizer algorithm

Year	Authors	Tasks
2021	Bass et al. [10]	Image Registration
2021	T. Kefato et al. [41]	Contrastive Learning Data Augmentation Self-Supervised Learning
2021	Najafi et al. [55]	Platform Semantic Similarity SemanticTextual Similarity Sentence Embedding
2021	Kamran et al. [40]	Disease Prediction Generation Image
2021	Zhang et al. [92]	Function Approximation

### Direct feedback alignment

A very deep 100-layer network can be trained with Direct Feedback Alignment (DFA). Furthermore, the reciprocal feedback assumption is replaced with a single feedback layer in DFA. Thus, DFA can be viewed as skipping the connections on a feedback path, allowing for more flexibility in the actual form of feedback connections. One of the first attempts of error-driven learning used directly coupled feedback routes. By skipping non-differentiable layers, the method can be utilized to deliver error signals. FA presumes a global feedback channel, which may be biologically untenable because of the single feedback layer’s enormous physical distance. The error signal is driven by the notion of “feedback alignment” in both FA and DFA. A layer cannot learn unless the layers above it are roughly aligned in the alignment step. Thus, FA and DFA are less effective optimization techniques. Replacing the backpropagation with a learning system with superior generalization performance is a more appropriate and biologically plausible path. In this scheme, the weights on a layer are updated by initially fixing the layer’s activation. Nonetheless, the theoretical findings of the negative descending direction have been inconclusive. Zhuge et al. [95] attempted to define the concept of integrity at the micro- and macro-levels. The model could highlight all the components corresponding to a specific salient object at the micro-level. At the macro-level, the model must discover all salient objects in the given visual scene. The novel Integrity Cognition Network (ICON) was designed to aid the integrity learning for salient object recognition. ICON was used to investigate three key components related to the learning of strong integrity features.

Ohana et al. [58] demonstrated the use of intrinsic noise of optical random projections to develop a differentially private DFA mechanism, which is the best approach for providing privacy-by-design training. Their theoretical study focused on the adaptive privacy technique, meticulously quantifying how optically random projection noise can cause differential privacy. According to test results, the proposed learning technique can achieve high end-task performance. Jinia et al. [50]investigated the extent to which DNN model training may be accomplished using a globally broadcast learning signal combined with local weight updates. A learning rule called the global error-vector broadcasting and a family of DNNs called the vectored nonnegative networks that use the learning rule were proposed. In this scheme, when the postsynaptic unit is activated, the learning rule generalizes the three-factor Hebbian learning by updating each weight using an amount proportionate to the inner product of the presynaptic activation and a globally broadcast error vector. Liu et al. [50] proposed learning the weight matrices in DFA in a backward manner by using the Kolen–Pollack learning methodology to increase training and inference accuracy of DNNs. Through training, the strategy can improve the learning accuracy and lower the gap between the parallel and serial training. Table 9 lists some of the tasks that commonly use the DFA optimizer algorithm.

**Table 9:** Some common related works for using Direct Feedback Alignment optimizer algorithm

Year	Author	Tasks	Datasets
2021	Zhuge et al. [95]	Object Detection Salient Object Detection	PASCAL-S
2020	Ohana et al. [58]	Image Classification	Fashion-MNIST
2020	Jinia et al. [39]	Image Classification	CIFAR-10 MNIST
2021	Liu et al. [50]	Image Classification	CIFAR-10 MNIST

### Layer-wise adaptive rate scaling (LARS)

Layer-Wise Adaptive Rate Scaling (LARS) is a technique for large-batch optimization. LARS differs from other adaptive algorithms, such as Adam or RMSProp, in two ways. First, LARS employs a separate LR for each layer rather than each weight. Second, aimed at improving the management of training pace, the size of the update is adjusted with respect to the weight norm. Goyal et al. [16]investigated whether self-supervision can be successfully implemented when large models are trained on non-curated images with no supervision. The model with 1.3 billion parameters and trained on 1 billion random images with 512 GPUs achieved 84.2% accuracy, exceeding the best self-supervised pre-trained model by 1%. This finding demonstrates that self-supervised learning can be implemented in real-world settings. According to Chen et al. [16], development was possible without requiring specific architectures or a memory bank. The major components of their framework were thoroughly explored as a means of determining the allowable contrastive prediction tasks in learning effective representations. Khosla et al. [42] proposed two different versions of the supervised contrastive loss to show which one performs the best. An accuracy of 81.4% on the ImageNet dataset with ResNet-200 was achieved, in which the value is 0.8% higher than the best value recorded for this architecture. On the other datasets and two ResNet variations, the cross-entropy was consistently surpassed. The loss has the features of resisting natural corruption, and it is more stable when handling hyperparameter settings, such as optimizers and data augmentations. Table 10 shows some of the tasks that commonly LARS optimizer algorithm.

**Table 10:** Some common related works for using LARS optimizer algorithm

Year	Authors	Tasks	Datasets
2020	Khosla et al. [42]	Contrastive Learning Data Augmentation	ImageNet ImageNet-C
2020	Chen et al. [16]	Image Classification	CIFAR-10 ImageNet Oxford 102 Flower
2021	Goyal et al. [33]	Image Classification	ImageNet COCO Places205 iNaturalist

### Adfactor

Adfactor is a stochastic optimization method based on Adam; it uses less memory while maintaining the empirical benefits of addictiveness. This algorithm is accomplished by ensuring that the squared gradient accumulator’s factored representation is constant across training steps. The Adfactor technique can reconstruct a low-rank approximation of the exponentially smoothed accumulator at each training step, which is a better optimal approach compared with the generalized Kullback–Leibler divergence. In the Adfactor method, the moving averages of the rows and columns sums of the squared gradients are tracked for the matrix-valued variables. Berkeley et al. [45] presented two strategies to improve transformer efficiency. The dot-product attention was replaced with locality-sensitive hashing to reduce the model complexity. Furthermore, reversible residual layers instead of normal residuals were used, allowing for the activations to be stored in the training phase only once rather than N times, where N is the number of layers. The Reformer is comparable with the transformer models in terms of performance, but it is significantly faster on extended sequences. Table 11 shows some of the tasks that commonly use the Adfactor optimizer algorithm. Xue et al. [84] presented byte-level models that are competitive with token-level models. The byte-level models are highly robust to noise, and they perform suitably on tasks that are sensitive to spellings and pronunciations. As part of the authors’ contribution, a new set of pre-trained byte-level transformer models based on the T5 architecture was released.

**Table 11:** Some common related works for using Adfactor optimizer algorithm

Year	Authors	Tasks	Datasets
2020	Berkeley et al. [45]	• Image Generation • Language Modelling	ImageNet Natural Questions
2021	Xue et al. [84]	• Natural Language Inference	N/A

### AMSGrad

**Fig. 12:**
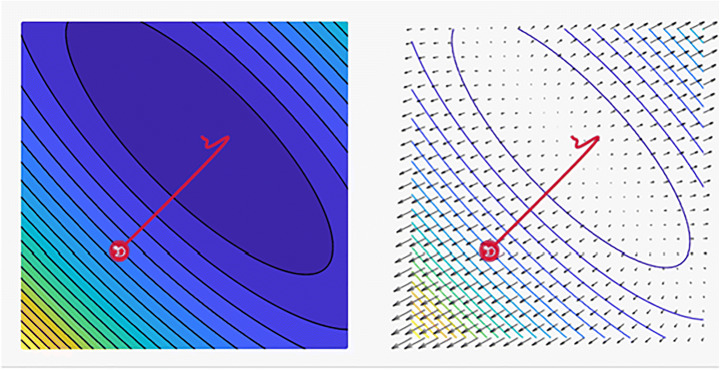
A plot of the loss reveals distinct properties for the Amsgrad optimizer with its style of convergence by ensmallen visualization tool, where the steps that the optimizer takes plotted in red. The global minimum is (1, 3) and the optimizer minimum is (1.024, 2.975) [28]

AMSGrad is a stochastic optimization method aimed at solving a problem by utilizing Adam-based optimizers. AMSGrad updates the parameters by using the maximum of previously squared gradients rather than the exponential average as shown in Fig. 12. Lim et al. [47] proposed a non-asymptotic analysis for the tamed unadjusted stochastic Langevin algorithm (TUSLA). Non-asymptotic error bounds were established for the TUSLA algorithm in Wasserstein-1 and Wasserstein-2 distances. The latter result allowed for the further derivation of non-asymptotic estimates in relation to the expected excess risk. Table 12 presents some of the tasks that commonly use the AMSGrad optimizer algorithm. Wang et al. [78] proposed a new motivation for designing the proximal function of adaptive algorithms called Marginal Regret Bound Minimization. On this basis, a class of adaptive algorithms that not only can achieve marginal optimality but also potentially converge much faster than any existing adaptive algorithms in the long term was proposed. The superiority of the class of adaptive algorithms was proven theoretically and empirically by performing experiments in DL.

**Table 12:** Some common related works for using AMSGrad optimizer algorithm

Year	Author	Tasks	Datasets	Metrics
2019	Lim et al. [47]	Applications to neural networks with relu activation function	N/A	N/A
2021	Wang et al. [78]	Classification Task	CT scan dataset	AUC = 0.90% ACC = 82.9%Sensitivity = 0.81%

### Gravity

Gravity is a kinematic approach to optimization based on gradients, i.e., a new approach to gradient-based optimization. Introduced by Bahrami et al. [8], this scheme describes how parameters can be changed to lower the DL model’s loss. Three intuitive hyperparameters for the optimal values were proposed. In addition, a moving average option was presented. Five typical datasets were trained on two VGGNet models with a batch size of 128 for 100 epochs to compare the performance of the Gravity optimizer with two common optimizers (Adam and RMS). According to Wang et al. [79], SwingBot is a robot that can learn the physical properties of a held object through tactile exploration. Tactile information is provided by two exploration actions (tilting and shaking), which are used to generate a physical feature embedding space. By using the embedding, a Swing Bot can anticipate the swing angle obtained by a robot conducting dynamic swing-up actions on a previously encountered object. Table 13 shows some of the tasks that commonly use the Gravity optimizer algorithm.

**Table 13:** Some common related works for using Gravity optimizer algorithm

Year	Authors	Datasets	Tasks
2021	Bahrami et al. [8]	CIFAR-10 MNIST CIFAR-100 Fashion-MNIST	Image Classification
2021	Wang et al. [79]	N/A	Self-Supervised Learning

Table 14 illustrates some common optimization algorithms tasks that help in detecting the best optimizer for a specific computer vision task. It is critical for anyone selecting an optimizer to identify the hyper parameters that may differ from one to the next. Model parameters are configuration variables that are internal to the model, and a model learns them on its own. The values of parameters can be estimated by optimization algorithms, such as gradient descent. Model hyperparameters are the learning rate for training a neural network. Detecting the initial values of hyparameters is essential for all types of optimizers that are shown in Table 14. The forthcoming section includes the proposed optimization algorithms to tackle two important challenges with two different types of datasets. The first one is the Seven Skin Cancer (SSC) detection based on ISIC dataset. While the second challenge is the utilization of Covid-19 CT and X-ray images extracted from COVIDx dataset. The two dataset are utilized to ensure the effect of optimizer algorithms with different types of medical images. Accordingly, we implement the two scinarios of images using the same hyperparameter values listed in Table 15. Furthermore, SGD and Adam optimizers achieved reliable and promising results comparing with other optimization algorithms.

**Table 14:** The most common optimization algorithms task

Year	Optimizer Name	Common Task used
1951	Stochastic Gradient Descent	Federated Learning Image Classification
1999	SGD with Momentum	Image Classification Object Detection
2000	Rung Kutta optimization	Object Detection
2011	AdaGrad	Language Modelling
2013	RMSProp	Image Classification
2014	Adam	Language Modelling
2014	Feedback Alignment	Object Detection Knowledge Distillation
2016	Deep Ensembles	Image Classification
2016	Direct Feedback Alignment	Image Classification Out-of-Distribution Detection
2017	LARS	Image Classification Self-Supervised Learning
2018	Adafactor	Question Answering
2019	AMSGrad	Time Series
2021	Gravity	Numerical Integration

**Table 15:** The default values for common optimization algorithms

Optimizer Name	Attribute	Default Value
AdaGrad	StepSize	0.01
	BatchSize	32
	Epsilon	1e-8
	MaxIterations	100,000
	Tolerance	Tolerance
	Shuffle	True
	Resetpolicy	True
	Exactobjective	False
Adam	Stepsize	0.001
	Batchsize	32
	Beta1	0.9
	Beta2	0.999
	Eps	1e-8
	Max_iterations	100,000
	Tolerance	1e-5
	Shuffle	True
	Resetpolicy	True
	Exactobjective	False
AdaMax	Stepsize	0.001
	Batchsize	32
	Beta1	0.9
	Beta2	0.999
	Eps	1e-8
	Max_iterations	100,000
	Tolerance	1e-5
	Shuffle	True
	Exactobjective	False
	Resetpolicy	True
AMSGrad	Stepsize	0.001
	Batchsize	32
	Beta1	0.9
	Beta2	0.999
	Eps	1e-8
	Max_iterations	100,000
	Tolerance	1e-5
	Shuffle	True
	Exactobjective	False
	Resetpolicy	True
Momentum SGD	Stepsize	0.01
	Batchsize	32
	Maxiterations	100,000
	Tolerance	1e-5
	Shuffle	True
	Updatepolicy	Momentumupdate()
	Decaypolicy	Decaypolicytype()
	Reset policy	True
	Exact objective	False
Nadam	Max_iterations	100,000
	Tolerance	1e-5
	Shuffle	True
	Reset policy	True

## Proposed method

In this section, we present two different types of medical images. One using colored skin cancer images, and the other using grayscale COVID-19 images. One of the most common diseases in the world is skin cancer. Given that the skin is the body’s largest organ, it is natural that skin cancer is the most prevalent type of cancer in humans [56]. DL reduces the need for feature engineering by learning and extracting meaningful features from raw data automatically. Many fields, particularly computer vision, have been transformed by DL. Furthermore, DL has recently achieved many successes in biomedical engineering. DL can reduce the need for feature engineering by learning and extracting meaningful features from raw data automatically. Many fields, particularly computer vision, have been transformed by DL. Furthermore, DL has recently achieved many successes in biomedical engineering, as shown in Table 15. Datta et al. [20] compared the performance of VGG, ResNet, InceptionResNetv2, and DenseNet architectures with and without the Soft-Attention mechanism while classifying skin lesions. The original network, when coupled with Soft-Attention, can outperform the baseline by 4.7% while achieving a precision of 93.7% on the HAM10000 dataset. Nadipineni et al. [54]. Mahboda et al. [51] developed a baseline classifier as the reference model without using any segmentation mask. On this basis, we used either manually or automatically created segmentation masks in both the training and test phases in different scenarios and investigated the classification performances. By using the International Skin Imaging Collaboration (ISIC) dataset from 2019, Hosny et al. [37] suggested a CAD system for skin lesions. However, this dataset is limited by many issues, including uneven classes. A multiclass SVM with a bootstrap-weighted classifier was then used. According to the image class, this classifier can adjust the weights. GoogleNet was also given a new class with a different quantity of unknown images, which were acquired from various sources for the training. Hameed et al. [35] suggested a multiclass and multilevel algorithm-based skin lesion classification system. With the suggested model, traditional ML and DL methods can be applied.

### Data augmentation

The data augmentation is conducted through affine transformations, and it involves the following elements: i) random brightness, ii) contrast changes, iii) random flipping, iv) random rotation, v) random scaling, and vi) random shear.

### Building deep learning model

**Fig. 13:**
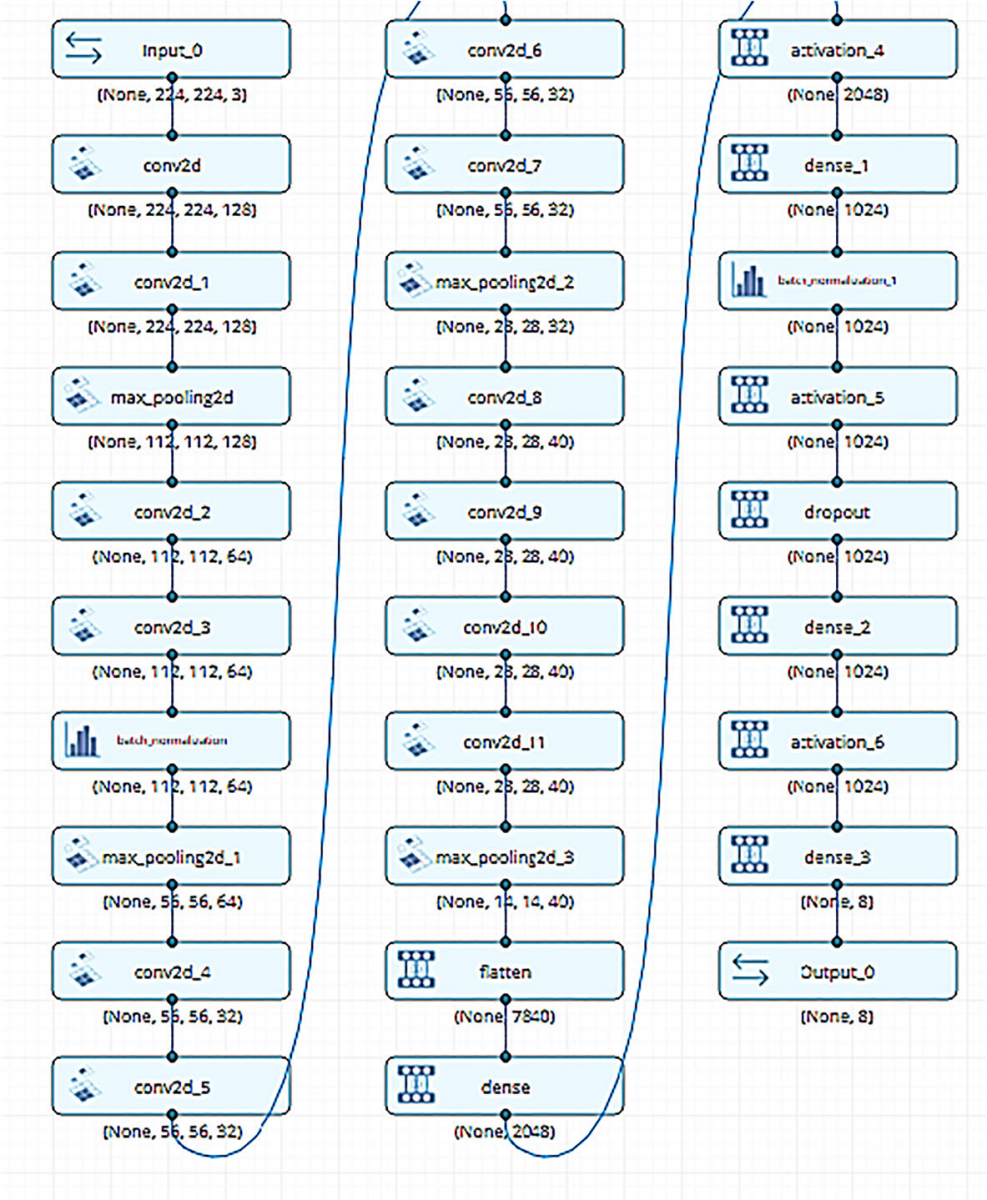
The proposed work model layers with input and output size

The Seven Skin Cancer (SSC) proposed model consists of CNN sequential layers, as shown in Fig. 13. The focus of this comparative survey on constructing an automated model for skin lesion classification is to enhance the model accuracy by incorporating the new methodologies. The accuracy is enhanced when new techniques are introduced into the equation. Although the CNN model has two layers, appropriate preprocessing, input, and training procedures can significantly improve the model accuracy. Data augmentation, image production via an adversarial generative network, and transfer learning can help to overcome the difficulty of training with a small dataset. Some academics rely on private datasets from the Internet. However, the required dataset is not available, and it is even more difficult to duplicate the findings and outcomes. Furthermore, the image selection from the Internet may be biased. Another key issue in this subject is the production of large public image collections containing photographs that can fully represent the world’s inhabitants to eliminate racial bias as shown in Fig. 13. Discrimination based on race and gender must be considered. For people from underrepresented gender or ethnicity, AI discrimination means that models and algorithms have failed to produce optimal results. In most current datasets, skin lesions on light-colored skin are the most apparent.

Data augmentation, image production via an adversarial generative network, and transfer learning could all help with the difficulty of training with a small dataset. Some academics rely on private datasets from the internet. Because the dataset is not available, it is more difficult to duplicate the findings and outcomes, and the image selection from the internet may be biased. Another key issue in this subject is the production of large public image collections containing photographs that are as representative of the world’s inhabitants as possible in order to eliminate racial bias. Consider discrimination based on race and gender. For people of an under-represented gender or ethnicity, AI discrimination means that models and algorithms fail to produce optimal results. In most current datasets, skin lesions on light-colored skin can be seen.

### Results

The SSC proposed model is applied to the ISIC dataset for skin cancer detection. Model evaluation is a core stage of measuring the performance of a model. In the following section, we compare the three optimizers (SGD, RMSProp, and Adam) commonly used for image classification tasks.

#### Datasets

**Fig. 14:**
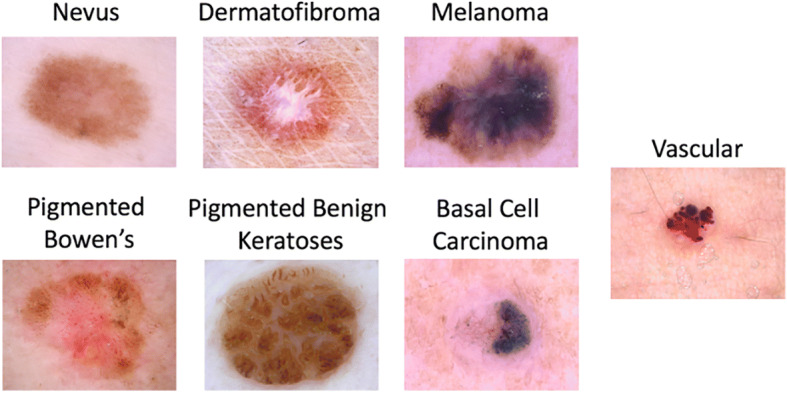
Random sample from seven classes of ISIC dataset

**Fig. 15:**
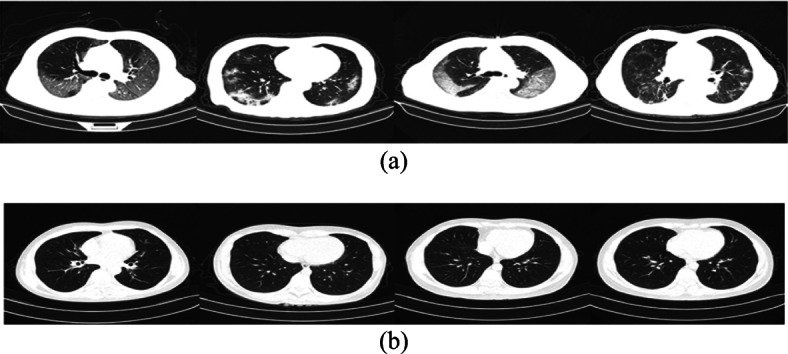
Example chest CT images from the COVIDx-CT dataset, (**a**) COVID-19 cases, and (**b**) Normal cases

This research applied the ISIC dataset [15] and COVIDx dataset [88] to review and evaluate a well-known dataset extracted from both skin cancer colored images and Covid-19 CT grayscale images, as shown in Figs. 14 and 15. The ResNet (50) model was pre-trained using the ISIC dataset, which contains 2594 images. The ISIC dataset covers seven classes. The work was written in CUDA and ran on a GPU. Using a GPU helps to sufficiently manage the voluminous training data while keeping the model error rate low. The SSC proposed model’s final three layers (completely connected, softmax layer, and classification layer) were eliminated and replaced with the new three algorithms. The preceding three layers of the pre-trained ResNet (50) were built to classify 1000 classes, but only seven classes (melanoma, melanocytic nevus, basal cell carcinoma, actinic keratosis, benign keratosis, dermatofibroma, and vascular lesion) were needed in the proposed work.

COVID-19 has infected over 1.3 million people around the world and caused the deaths of over 106,000 people. Inefficiency and a lack of diagnosis are two major roadblocks to regulating the progression of this disease. We compared different types of optimizations with another dataset that we called COVIDx CT, a benchmark CT image dataset derived from a variety of sources of CT imaging data currently comprising 104,009 images across 1489 patient cases. We used a sample of 13,413 cases that were divided into two class labels; 7395 infected COVID-19 cases and the remaining 6018 were not infected or normal cases. The figure shows samples of chest CT images with COVID-19 CT cases and normal cases.

The proposed model’s reliability was assessed by considering several performance indicators, including sensitivity, specificity, precision, negative predictive value, false-positive rate, false discovery rate, accuracy, F-score, and Matthews Correlation Coefficient. These measures can be computed on the basis of the following Eqs. (10–19) [72]:

11 $$Recall=\frac{\mathrm{TP}\ }{\ \left(\mathrm{TP}+\mathrm{FN}\right)}$$

12 $$\mathrm{Specificity}=\frac{\mathrm{TN}\ }{\left(\mathrm{FP}+\mathrm{TN}\right)}$$

13 $$\mathrm{Precision}=\frac{\mathrm{TP}\ }{\left(\mathrm{TP}+\mathrm{FP}\right)\ }$$

14 $$\mathrm{NPV}=\frac{\mathrm{TN}\ }{\left(\mathrm{TN}+\mathrm{FN}\right)}$$

15 $$\mathrm{FPR}=\frac{\ \mathrm{FP}\ }{\ \left(\mathrm{FP}+\mathrm{TN}\right)}$$

16 $$\mathrm{FDR}=\frac{\mathrm{FP}}{\ \left(\mathrm{FP}+\mathrm{TP}\right)}$$

17 $$\mathrm{FNR}=\frac{\mathrm{FN}\kern0.5em }{\left(\mathrm{FN}+\mathrm{TP}\right)}$$

18 $$\mathrm{Accuracy}=\frac{TN+ TP}{TP+ FP+ TN+ FN}$$

19 $$\mathrm{F}1-\mathrm{score}=\frac{2\mathrm{T}\mathrm{P}}{2\mathrm{T}\ \mathrm{P}+\kern0.5em \mathrm{F}\ \mathrm{P}+\mathrm{F}\ \mathrm{N}}$$

20 $$\mathrm{MCC}=\frac{\mathrm{TP}\ast \mathrm{TN}-\mathrm{FP}\ast \mathrm{FN}\ }{\mathrm{Sqrt}\left(\left(\mathrm{TP}+\mathrm{FP}\right)\ast \left(\mathrm{TP}+\mathrm{FN}\right)\ast \left(\mathrm{TN}+\mathrm{FP}\right)\ast \left(\mathrm{TN}+\mathrm{FN}\right)\right)}$$

where TP, FP, FN, and TN refer to a true positive, false positive, false negative, and true negative, respectively.

#### Optimizer algorithms

Optimizers are methods or strategies for lowering losses by altering the neural network’s features, such as weights and LR. Optimizers are used to address the optimization problems by minimizing the function. The main metrics values with the ISIC dataset are illustrated in Table 16.

**Table 16:** A related works for Skin cancer diagnosing on ISIC dataset

Year	Authors	Model	Task	Metrics values
2021	Datta et al. [20]	Soft Attention	Image Classification Lesion Classification	ACC = 93.40% AUC = 98.40% Precision = 93.70%
2020	Nadipineni et al. [54]	Skin lesion classification	Data Augmentation Lesion Classification	N/A
2020	Mahbodet al. [51]	CNN Classification Model	Skin Lesion Classification	N/A
2020	Hosny el al. [37]	Transfer learning model	Classifying the challenging dataset ISIC2019	ACC = 98.70% AUC = 95.60% Precision = 95.06%
2020	Hameed et al. [35]	K-means, transfer learning, Augmentation.	Skin lesions Classification	N/A
2020	Zhang el al. [91]	Optimized algorithm for weight selection	Applying genetic algorithm	

##### SGD optimizer

**Fig. 16:**
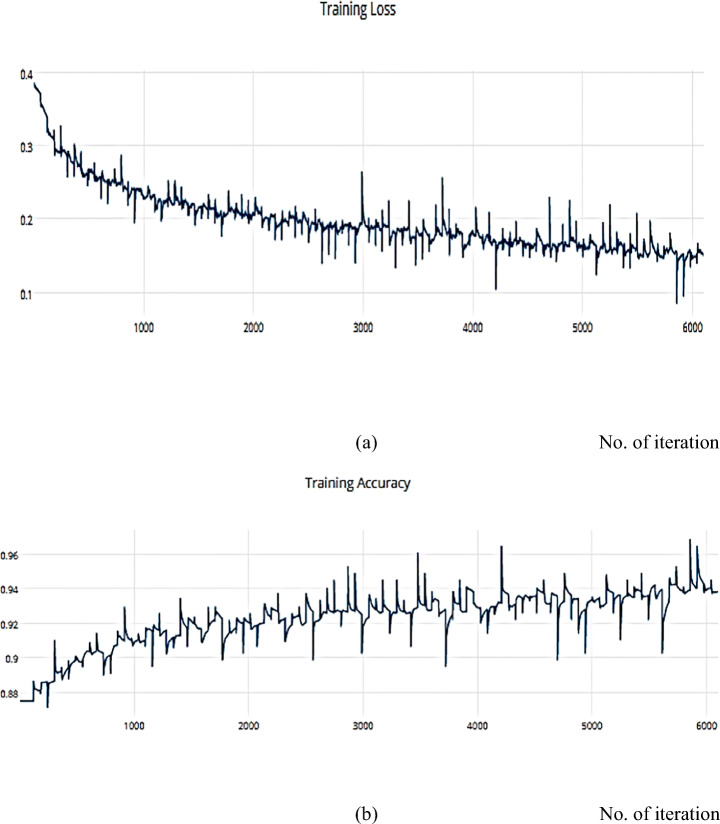
**a** The Training Loss, **b** The Training accuracy of the proposed model based on SGD optimizer on ISIC dataset

**Fig. 17:**
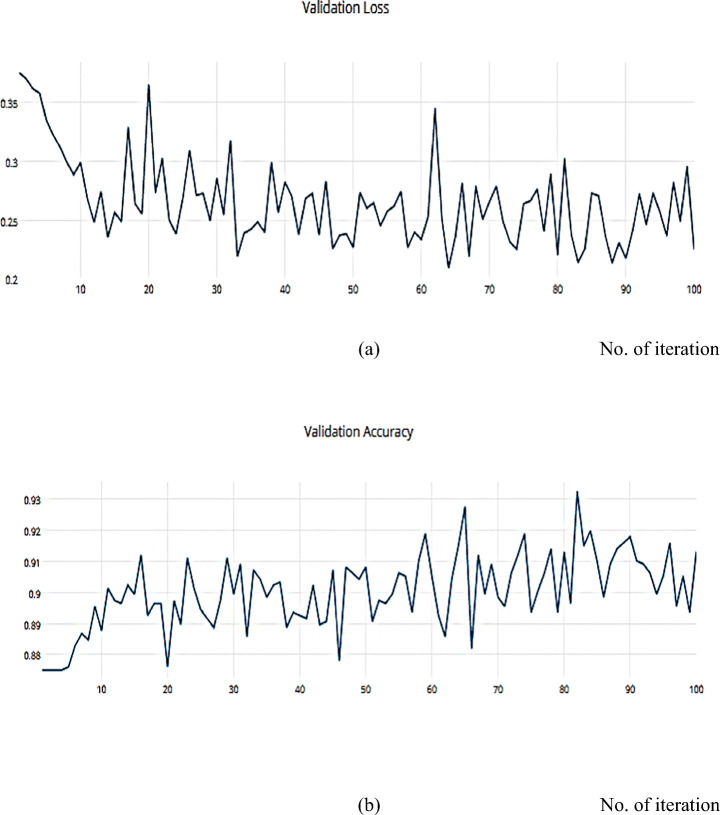
**a** The Validation Loss, **b** The Validation accuracy of the proposed model based on SGD optimizer on ISIC dataset

Gradient Descent has the disadvantage of requiring voluminous memory to load the entire dataset of n-points at a given time to compute the derivative of the loss function. Nonetheless, some of the disadvantages of the SGD algorithm can be alleviated. Nesterov Momentum is a slight variation of the normal gradient descent, and it can significantly speed up the training and improve the convergence. We applied the SGD optimizer to the ISIC dataset and achieved an accuracy measure metric of 0.9445%, as shown in Figs. 16 and 17.

##### RMSProp optimizer

**Fig. 18:**
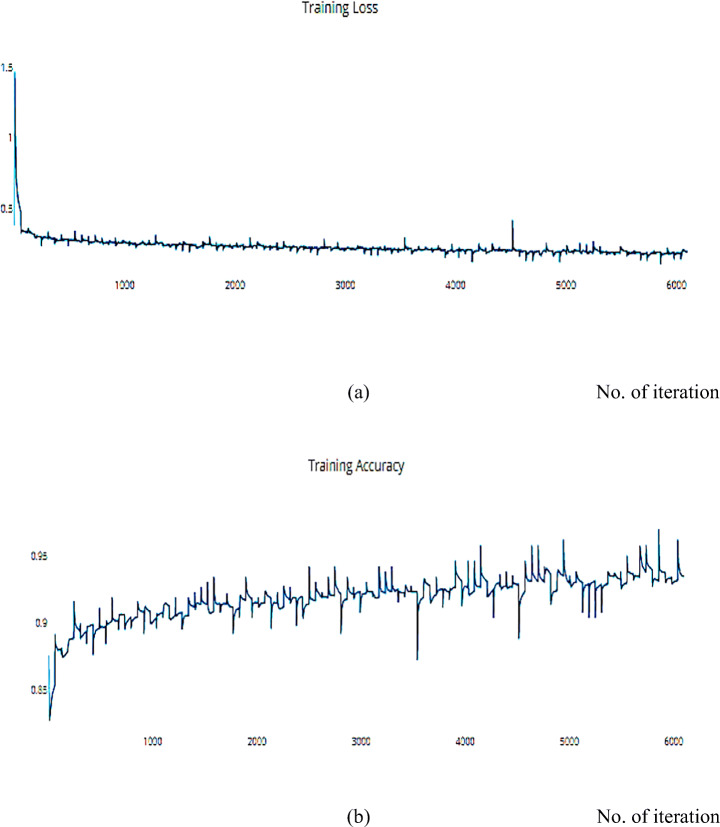
**a** The Training Loss, **b** The Training accuracy of the proposed model based on RMSProp on ISIC dataset

**Fig. 19:**
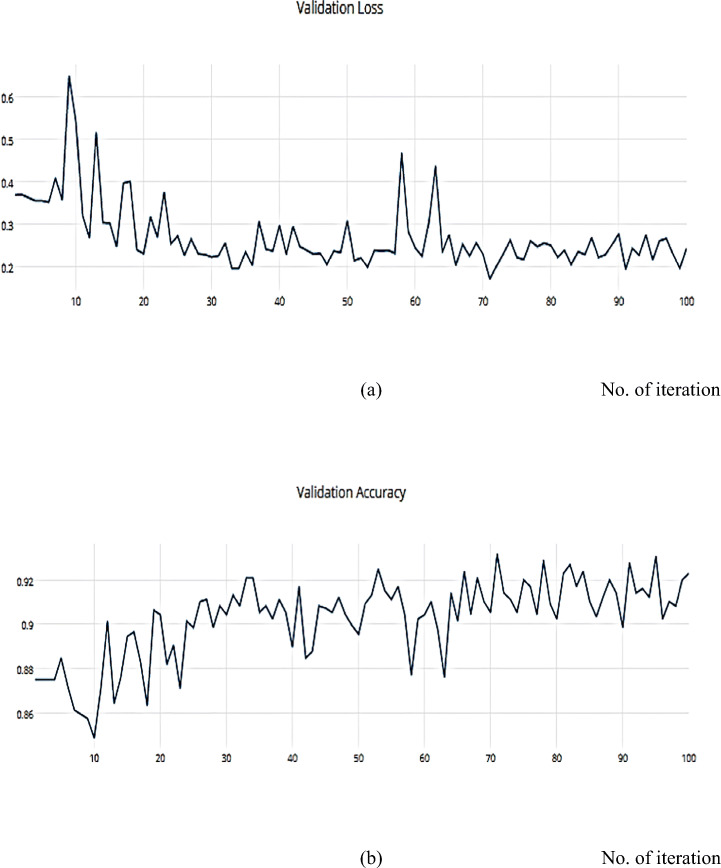
**a** The Validation Loss, **b** The Validation accuracy of the proposed model based on RMSProp on ISIC dataset

The RMSProp optimizer aids in various computer vision tasks by utilizing leaky averaging, which it shares with momentum. Figures 18 and 19 show the accuracy measure metrics in relation to the effect RMSProp.

##### Adam

**Fig. 20:**
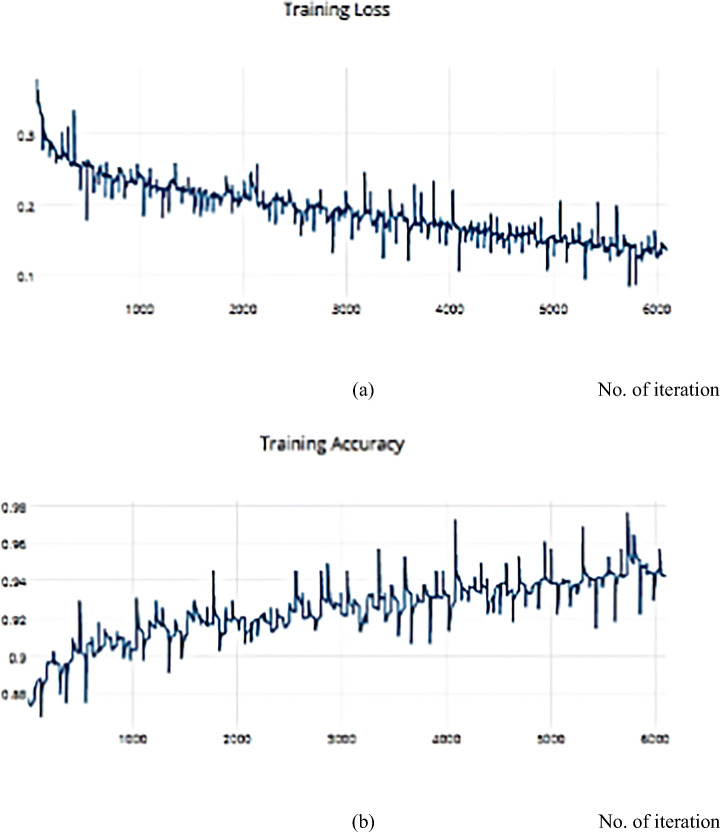
**a** The Training Loss, **b** The Training accuracy of the proposed model based on Adam optimizer on ISIC dataset

**Fig. 21:**
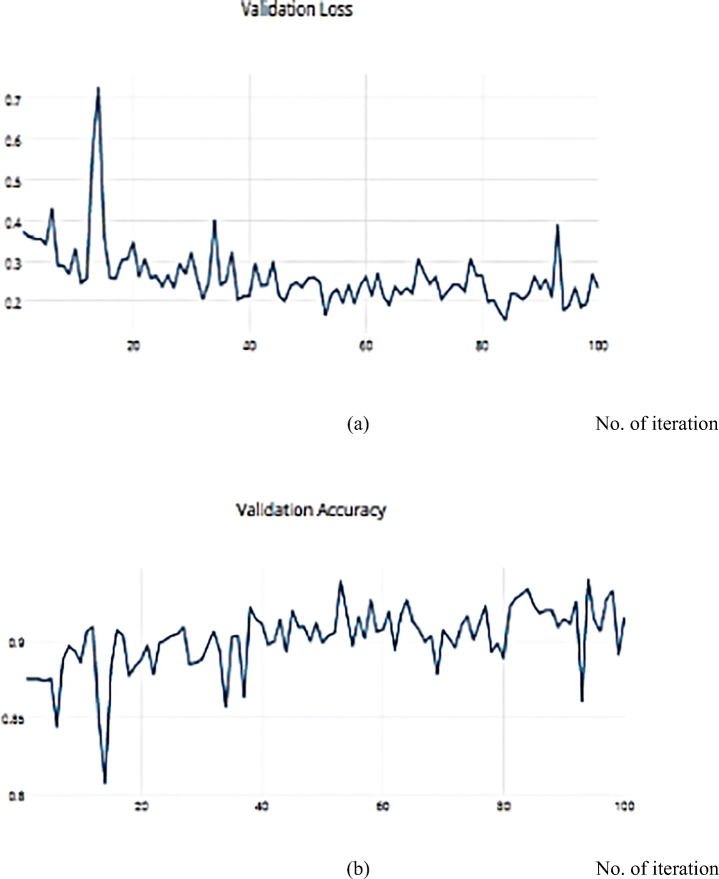
**a** The Validation Loss, **b** The Validation accuracy of the proposed model based on Adam optimizer on ISIC dataset

Adam can be viewed as a combination of RMSprop and SGD, with the addition of momentum. For each parameter, Adam calculates the adaptive LRs as investigated in Figs. 20, and 21 which describes the training, and validation loss and accuracy, respectively.

**Fig. 22:**
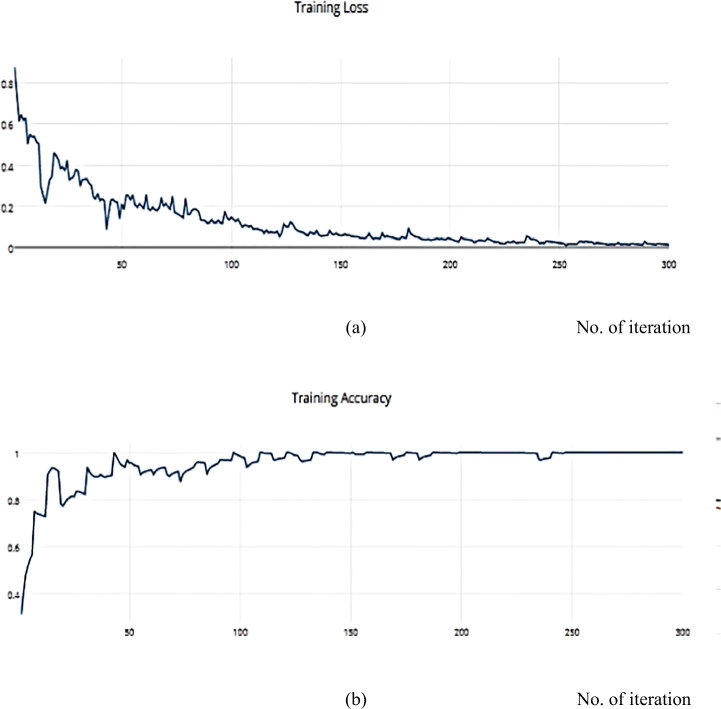
**a** The Training Loss, **b** The Validation accuracy of the proposed model based on Adam optimizer on COVIDx dataset

**Fig. 23:**
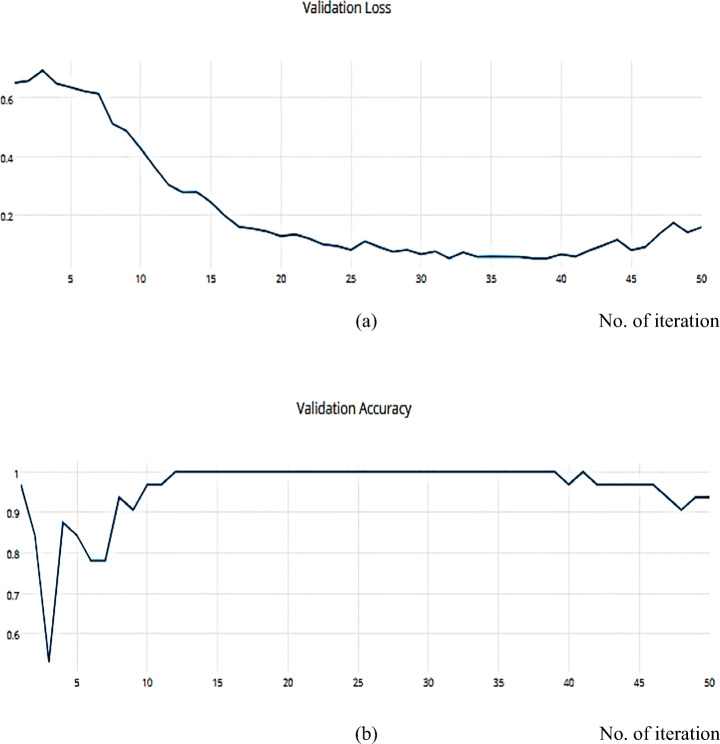
**a** The Validation Loss, **b** The Validation accuracy of the proposed model based on Adam optimizer on COVIDx dataset

**Fig. 24:**
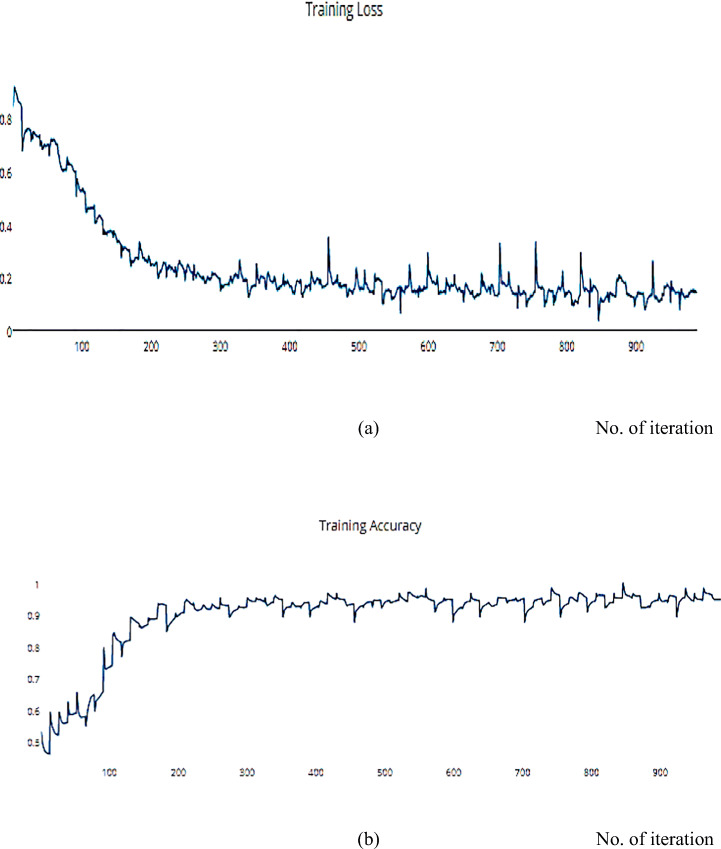
**a** The Training Loss, **b** The Training accuracy of the proposed model based on SGD optimizer on COVIDx dataset

**Fig. 25:**
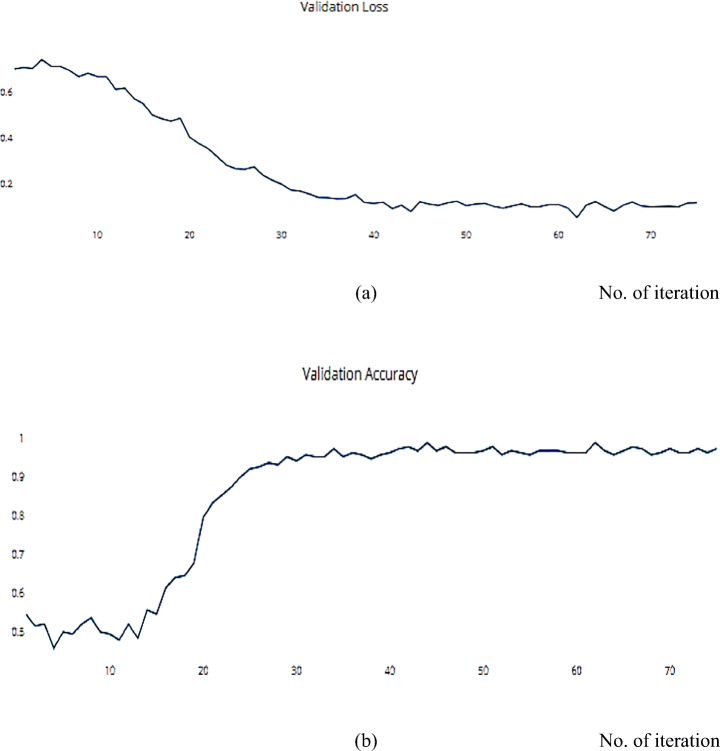
Note: This data is mandatory. Please provide

Compared with another dataset is a helpful way to decide and distinguish between different optimization algorithms. The SSC model is implemented on the COVIDX dataset and achieves a metric result that is converging with the ISIC dataset. For the previous implementation with ISCI, we decided to use the ADAM and SGD optimizers for this comparison. Figures 22 and 23 have the training and validation curves with the ADAM optimizer. Figures 24 and 25 show the training and validation curves with the SGD optimizer. Metric values are illustrated in Table 17. We utilised the subsampled COVIDx dataset and analysed the performance of the proposed algorithm using both Adam and SGD optimize as shown in Table 18. We found that slightly improved results were achieved. On the other hand, we plan to use other classifiers to monitor the performance of algorithms that may exhibit performance degradation. This behaviour results from the fact that the classifiers may still use the other features to provide an accurate performance even if one feature has declined. However, if the quality of every feature decreased, the algorithm’s performance would likewise have decreased. Therefore, every classifier acted appropriately when the sampling duration was long. Therefore, the ideal classifier to use in order to apply this technique should be one that delivers [14].

**Table 17:** The overall common metrics for all optimization algorithms with ISIC dataset

SGD Optimizer									
Sensitivity	Specificity	Precision	NPV	FPR	FDR	FNR	Accuracy	F-score	MCC
0.9507	0.9370	0.9480	0.9402	0.0630	0.0520	0.0493	0.9445	0.9494	0.8879
RMSprop Optimizer									
Sensitivity	Specificity	Precision	NPV	FPR	FDR	FNR	Accuracy	F-score	MCC
0.9133	0.9091	0.9199	0.9016	0.0909	0.0801	0.0867	0.9113	0.9166	0.8220
Adam Optimizer									
Sensitivity	Specificity	Precision	NPV	FPR	FDR	FNR	Accuracy	F-score	MCC
0.9773	0.9685	0.9700	0.9762	0.0315	0.0300	0.0227	0.9730	0.9737	0.9460

**Table 18:** The overall common metrics for training and testing stage with COVIDx dataset

Training Stage										
Metrics	Recall	Specificity	precision	NPV	FPR	FDR	FNR	Accuracy	F-score	MCC
Adam	0.9903	0.9854	0.9851	0.9905	0.0146	0.0149	0.0097	0.9878	0.9877	0.9757
SGD	0.9890	0.9853	0.9850	0.9892	0.0147	0.0150	0.0110	0.9871	0.9870	0.9742
Testing Stage										
Metrics	Recall	Specificity	precision	NPV	FPR	FDR	FNR	Accuracy	F-score	MCC
Adam	0.9953	0.9862	0.9859	0.9954	0.0138	0.0141	0.0047	0.9907	0.9906	0.9814
SGD	0.9933	0.9860	0.9857	0.9935	0.0140	0.0143	0.0067	0.9896	0.9895	0.9793

This research presents a comparative survey of several optimization algorithms and a comprehensive study of diagnosing skin cancer infection with deep CNN models. The selected available and known algorithms are described and then compared. The comparison of the skin lesion classification methods indicates that the problem formulations of each study vary slightly. The efficient melanoma detection process entails five core elements: data acquisition (collection), fine-tuning, selection of features, DL, and final model development. The first step involves the acquisition of data on skin cancer detection from publicly available benchmarks and non-listed and non-public databases, such as the melanoma detection images collected from the Internet.

## Conclusion

The survey was run by optimization algorithms such as AdaMax, SGD, Root Mean Square Propagation, Adaptive Gradient Algorithm, Namax, and Adam. Optimization algorithms are available and commonly used to solve complex problems. Then, a comprehensive survey was conducted, aiming to gain deeper insights into the different aspects of the algorithms. Among the optimization algorithms, results are better when trapping is prevented by local optimal solutions. The performance of AdaMax is superior among the selected algorithms in terms of numerical function optimization. DL makes intelligent decisions on its own and ultimately achieves a higher accuracy rate. The pre-trained DL models and handcrafted methods based on the DL approach have already shown promising results for the high-precision accuracy of melanoma detection. However, in this study, we highlighted the importance and effect of optimization algorithms to improve the accuracy of the applied medical image datasets with different challenges, such as skin cancer and COVIDx. We further highlight the location problem and how to tackle this problem to boost the performance of the algorithm with different applied classifiers and datasets. In the future, we plan to use it to monitor the performance of the algorithm with a sub-sampled dataset. In this way, it is possible to know which algorithm is extracting more information from the data.

## Data Availability

https://challenge.isic-archive.com/landing/2018/ https://github.com/ncbi-nlp/COVID-19-CT

## Code availability

Availble on Rquest.
